# Calibration of SB Brasil 2023 examiners: use of technologies associated with the In-Lux method

**DOI:** 10.1590/1807-3107bor-2025.vol39.045

**Published:** 2025-05-19

**Authors:** Raquel Conceição FERREIRA, Rafaela da Silveira PINTO, Claudiojanes dos REIS, Rosa Núbia Vieira de MOURA, Sara Oliveira AGUIAR, Andreia Maria Araújo DRUMMOND, Viviane Elisângela GOMES, Andrea Maria Eleutério de Barros Lima MARTINS

**Affiliations:** (a) Universidade Federal de Minas Gerais, School of Dentistry, Department of Social and Preventive Dentistry, Belo Horizonte, MG, Brazil.; (b) Faculdades Unidas do Norte de Minas – Funorte, Faculty of Health Sciences, Montes Claros, MG, Brazil.; (c) Universidade Federal de Minas Gerais, School of Dentistry, Departament of Dentistry, Belo Horizonte, Minas Gerais, Brasil.; (d) Universidade Estadual de Montes Claros – Unimontes, School of Dentistry, Montes Claros, MG, Brasil.

**Keywords:** Health Surveys, Observer Variation, Education, Distance, Calibration, Epidemiology

## Abstract

This methodological study presents the development and implementation of technological tools for the online training and calibration of dentists participating in the SB Brasil 2023 survey, using the Moodle® platform. The training and *in-lux* calibration process employed 10 and 25 sets of photographs, respectively. Conditions including dental crown status (dmft and DMFT indexes), caries consequences (pufa/PUFA index), malocclusion (canine relation, overjet, overbite, posterior crossbite, and Dental Aesthetic Index), and dental trauma were evaluated, based on WHO or SB Brasil 2010 criteria. Software was developed to record codes, automated calculations of agreement coefficients (overall percentage, score-specific, and simple and weighted kappa), and generate reports. Examiners were allowed to repeat calibration attempts until they achieved a minimum agreement (Kappa ≥ 0.61). In total, 1,513 examiners used the software, and 728 successfully completed the calibration with substantial or higher agreement across all conditions/indexes. Individual reports described discrepancies and monitored attempts. Occlusal conditions had the lowest percentages of almost perfect agreement and required more attempts to achieve calibration. The technological tools implemented in SB Brasil 2023 enabled online training and calibration, promoting consistency among examiners for fieldwork. These findings demonstrate the feasibility of remote strategies for calibration in epidemiological surveys, particularly in scenarios involving multiple geographically distributed examiners, with potential applications in other contexts and health areas.

## Introduction

Population-based oral health surveys estimate the prevalence of major oral health conditions, providing data to guide public policies and plan dental services effectively. These surveys are complex, costly, and require rigorous methodological standards to ensure unbiased and reliable results. A key challenge in such surveys is the involvement of multiple examiners, requiring standardized diagnostic criteria to ensure findings’ reliability, comparability, and validity.^1,2^

Since the 1990s, the World Health Organization (WHO) has recommended training and calibration protocols to standardize the interpretation and application of diagnostic criteria for obtaining dental index.^2,3^ The training familiarizes examiners with research protocols, diagnostic criteria, and assessment codes.^4^ At the same time, calibration compares their measurements to reference standards (validity) and evaluates consistency in and among examiners (intra- and inter-examiner reliability).^3,4^ The goal of calibration is to ensure uniform interpretation and application of diagnostic criteria, ensure examiners consistently adhere to a standard, and minimize variability among examiners.

Traditionally, calibration has been conducted through in vivo clinical examinations, which simulate actual data collection conditions. However, this method has logistical and ethical challenges,^4-6^ including participant recruitment, repeated examinations, addressing low prevalence conditions, and the extensive time and infrastructure required.^4-7^ Furthermore, in vivo calibration may not always be feasible due to resource limitations or specific population challenges, such as young children, individuals with disabilities, or older adults.^4^

The use of photographic records for calibration—known as the in-lux method—has been proposed to overcome these limitations. This approach uses high-quality photographs representing various dental conditions. First implemented in the 2010 Brazilian Oral Health Survey (SB Brasil 2010) to assess examiner reliability for dental trauma and fluorosis,^8^ the *in-lux* method has since been successfully applied in other surveys, including a regional survey in Minas Gerais^9^ and a schoolchildren oral health survey in Pelotas, Brazil,^10^ mainly to evaluate low-prevalence conditions. Also, previous studies have demonstrated the validity of photographs to evaluate dental caries,^4,7,11^ and plaque scoring system.^12^

A comparative analysis of the in vivo and *in-lux* methods showed that both can effectively identify examiners’ reliability to assess dental caries (DMFT index) and malocclusion (DAI), with the *in-lux* method offering greater feasibility in resource-constrained settings or when involving many examiners.^7^ The *in-lux* method provides several advantages: it enables the calibration of many examiners in a shorter timeframe, reduces logistical costs, minimizes ethical concerns for participants, and ensures consistent assessments across geographically dispersed examiners. Additionally, it facilitates shorter intervals between calibration and data collection and enhances reliability assessment for low-prevalence conditions.^7,9^

Reporting agreement metrics among examiners is essential for validating survey results. The WHO recommends global agreement percentages and simple kappa statistics for consistency evaluation. The weighted kappa statistic is preferred for ordinal measures, as it accounts for the degree of disagreement, penalizing more severe misclassifications.^13^ Traditionally, spreadsheet software calculates agreement coefficients manually during calibration exercises. This approach can be time-consuming and may contribute to fatigue among participants, especially when repeated attempts are necessary due to unsatisfactory results.

The integration of new technologies could streamline this process by enabling examiners to record results faster and calculate agreement coefficients more efficiently. Furthermore, these technologies could facilitate repeated calibration exercises with greater ease. During the COVID-19 pandemic, the 2020 Brazilian National Epidemiological Survey (here called SB Brasil 2023) was suspended, highlighting the need for innovative solutions to ensure continuity in research. In this context, alternative strategies were developed, adapting training and calibration processes to online formats mediated by digital technologies. Given prior evidence and experiences with the *in-lux* method, the SB Brasil 2023 survey adopted this approach, incorporating electronic technologies to optimize data collection, storage, and analysis.^7,9^ This article describes the development and implementation of calibration software and a data management system that supported the *in-lux* calibration method used in the SB Brasil 2023 survey.

## Methods

Methodological study on the development and implementation of technological tools for the training and calibration of examiners in the SB Brasil 2023, coordinated by the Brazilian Ministry of Health, with data collection taking place in 2023 and 2024. The development and testing of the technological tool occurred from June 2020 to August 2021.

### Training and calibration of the field team

The examiners were dentists working in Primary Health Care within the Unified Health System (SUS), who performed oral exams at participants’ homes in a probabilistic sample of participants at the index-ages of 5 and 12 years, and age groups 15–19, 35–44, and 65–74 years, as defined by the WHO, to evaluate oral health conditions among children, adolescents, adults, and elderly individuals, respectively. The exams were conducted following codes and criteria defined by WHO or those adopted in SB Brasil 2010, aiming to maintain the historical data series for oral health surveillance, as detailed in the technical project.^14^ This process was monitored by field supervisor researchers in each region of the country, defined as Local References.

The theoretical-practical training was conducted online, with synchronous and asynchronous activities, using a virtual learning environment built on the Moodle® platform. The theoretical training on the research methodology, codes, and criteria for assessing oral health conditions was conducted for a minimum of 16 hours. The codes and criteria for the assessed conditions were described and illustrated with photographs in video lessons, in addition to being detailed in the Examiner’s Manual.

The practical training involved one exercise and calibration. In these two activities, the *in-lux* method was adopted, which uses photographs of the mouth and teeth to replicate the situation the examiner would encounter in the field.^7^ Ten sets of photographs were used for the training and 25 for the calibration of the examiners, aiming to encompass the diversity of conditions encountered in fieldwork. The photographs were organized into separate PDF files. Each photograph or set of photographs was labeled with the term “volunteer,” numbered sequentially (e.g., volunteer 1, volunteer 2). A Nikon camera model D750, Macro Lens 100 mm, and Nikon circular and twin flashes were used to capture the photographs by a professional photographer who had completed dental photography courses totaling 200 hours of training. Most of the volunteers were patients undergoing treatment at the dental clinics of the Faculty of Dentistry at UFMG. However, due to the COVID-19 pandemic and the interruption of clinical services, photographs of children were also obtained in other locations, as they had not yet been collected in sufficient numbers. The photographs for training and calibration of trauma and clinical consequences of dental caries were obtained from the archives of professionals and professors from the dental trauma extension project at the Faculty of Dentistry at UFMG.

In the photographs, the examiners assessed crown caries in deciduous and permanent teeth for the evaluation of the dmft/DMFT (Decayed, Missing, and Filled Teeth) index, clinical consequences of untreated caries using the pufa/PUFA (pulpal involvement, ulceration, fistula, and dentoalveolar abscess) index, occlusal condition in deciduous dentition (canine relation, overbite, overjet, and posterior crossbite), conditions for obtaining the Dental Aesthetic Index (DAI) and dental trauma.

Each set of photographs included 12 images of the entire mouth, with frontal, occlusal, and lateral shots (in Maximum Habitual Intercuspation) to assess dental crown caries in all deciduous and permanent teeth and occlusal conditions. For the crown condition of each of the 20 deciduous teeth, a total of 200 records were made during training and 500 records during calibration. For the 28 permanent teeth, these numbers were 560 and 700 records, respectively. Some of these photographs were taken with a WHO-type periodontal probe positioned to measure the occlusal conditions, with overbite in the deciduous dentition (defined as increased when the overbite measurement in millimeters was > 2 mm), and the following conditions assessed for the DAI: incisal diastema (space, in millimeters, between the two upper central incisors), maxillary anterior misalignment (largest irregularity, in millimeters, between upper incisors), mandibular anterior misalignment (largest irregularity, in millimeters, between lower incisors), maxillary anterior overjet (distance, in millimeters, from the incisal edge of the most prominent upper incisor to the vestibular surface of the corresponding lower incisor), mandibular anterior overjet (measurement, in millimeters, of mandibular protrusion), and anterior vertical open bite (measurement, in millimeters, of the distance between the upper and lower incisal edges). For the assessment of clinical consequences of dental caries and dental trauma, individual photographs with marked teeth to be evaluated were used. For occlusal conditions, clinical consequences of dental caries and dental trauma, only one record was made per volunteer, meaning 10 records during training and 25 records during calibration.

### Standard Examination

The sets of photographs were evaluated by a team of five researchers to define the codes for each assessed condition and volunteer/tooth, just as was done during the training and calibration by the examiners. The researchers were graduates in Dentistry, with prior experience in epidemiological surveys, and underwent theoretical training prior to evaluating the photographs using the same material provided to the examiners: the Examiner’s Manual and the video lessons about the oral health conditions addressed in the training and calibration. Each researcher independently assigned the codes, recording them in a form on Google Forms®. Meetings were held to review the codifications. In case of disagreements, the codes and criteria were discussed based on the material provided until a consensus was reached, assigning a final code to each tooth or volunteer. This consensus outcome was referred to as the standard examination, which was considered for determining the agreement among the examiners in the study. In the context of *in-lux* calibration, the reference standard served as the benchmark examiner, an experienced assessor assumed to be error-free or nearly so.^15^ Data from training and calibration exercises were used to evaluate the level of agreement between the examiners and the benchmark. This process assessed inter-examiner agreement, requiring all examiners to achieve reproducible results compared to the reference standard. The consensus outcome was recorded in the data management system as the “reference standard” and used for calculating agreement coefficients.

After the consensus, the results of the standard examination for calibration showed that the volunteers had an average dmft of 3.36, with 56% having dmft > 1. Thirteen children had at least one decayed tooth, with an average of 2.24 decayed teeth. Filled teeth were identified in 10 children, with an average of 1.08 per child. In the permanent dentition, the volunteers had at least one decayed tooth, with an average of 2.24 decayed teeth. Additionally, 80% had one or more filled teeth (average of 6.68), and 52% had missing teeth due to caries (average of 2.76). Regarding the pufa/PUFA index, 32% of the teeth showed no clinical consequences of caries, while 4% had dentoalveolar abscesses, 24% had fistulas, 32% had pulpal involvement, and 8% had ulcers. As for dental trauma, the distribution was: no trauma (8%), treated fracture (16%), enamel fracture (20%), enamel and dentin fracture (24%), fracture with pulpal involvement (12%), tooth loss due to trauma (8%), other damage (8%), and one record of an examination not performed due to the absence of incisors. In the DAI index, the distribution was: no malocclusion (44%), defined malocclusion (32%), severe malocclusion (12%), and very severe malocclusion (12%). In the deciduous dentition, based on the canine relation evaluation, 76% of the children were class I, 16% were class II, and 4% were class III. For four children, code 9 was assigned due to inability to evaluate. Regarding overbite, 36% had a normal relationship between the incisors, 8% had reduced bites, 28% had deep bites, and for 28% of the children it was not possible to evaluate. As for overjet, 40% had a normal relationship, 28% had an increased bite, and 92% did not have posterior crossbite.

### Data recording, data management system recording, and report generation

For the recording of the codes and criteria for the conditions assessed by the examiners in the research, a software integrated into a data management system, named SC Brasil, was developed. This system enabled the research team to issue reports for monitoring the calibration process. The software development considered functionalities designed to meet the specific needs of SB Brasil 2023, as described in Table 1.

**Table 1:** Functionalities of the data recording software for training and calibration of examiners in SB Brasil 2023, and the data management and reporting system.

Data recording software for training and calibration of SB Brasil 2023 examiners
• restricted access granted through individual registration as an examiner for SB Brasil 2023, with subsequent access using login and password,
• entry of records according to photographs produced for training and in-lux calibration: records of all teeth (dental caries) or a single record per volunteer depending on the evaluated condition,
• entry of records of the conditions evaluated for 10 volunteers during the training phase and 25 during the calibration phase, with pre-selection of the volunteer to be evaluated,
• entry for condition recording with pre-registered categories identical to those described in the Examiner’s Manual and video lessons, or with typing error control for responses with whole numbers. For example, for recording the condition of the dental crown in deciduous teeth, the pre-registered options were A: Healthy, B: Decayed, C: Filled, with caries, D: Filled, without caries, E: Missing due to decay, F: Sealant, G: Bridge support or crown/implant. For recording the incisal diastema measurement in millimeters, only whole numbers starting from 0 could be entered,
• record entries organized following the same sequence proposed for conducting fieldwork examinations. For instance, dental condition recording began with tooth 17 and proceeded sequentially to 27; then continued from 37 to 38,
• offline functionality ensuring data entry even in areas with limited connectivity,
• records saved as data were entered, allowing subsequent submission once internet access was available,
• compatibility with Android® operating system, due to the configurations of the Mobile Data Collection Device provided by the Brazilian Institute of Geography and Statistics for data collection,
• availability of a Desktop version with the same features as the mobile app, facilitating use in all examiners’ work environments,
• possibility of multiple-record entries for the same volunteer by the examiner, allowing repetition of records when an adequate agreement coefficient was not achieved on the first attempt,
• ability to edit records while typing errors were detected, limited to the moment of submission, after which editing was no longer permitted,
• calculation of the DAI index score based on the weighting of assessed malocclusion conditions, followed by classification of malocclusion according to the methodology of Jenny and Cons (1996) (16),
• exporting entered data to the data management system.
**Data management and report generation**
• mechanisms to ensure the integrity and security of recorded data,
• restricted access to examiner reports under their responsibility, according to state/region, via login and password by Local References, and unrestricted access to all examiners’ results by the SB Brasil 2023 coordination team,
• automated calculation of agreement coefficients between each examiner’s records and the “standard examination”,
• generation of reports to monitor training, based on an individual analysis of each examiner’s results by condition and attempt, as well as grouped reports for all examiners under a given Local Reference’s responsibility. In the individual analysis, the report includes a cross-tabulation between the examiner’s records and the “standard examination,” facilitating the understanding of disagreements and guiding the reinforcement of codes and criteria for a new attempt. The reports also provide a summary of agreement coefficients for each epidemiological index of examiners grouped by municipality and region. Examiners were identified by codes generated upon software registration, as well as by name, municipality, and state,
• green indicators for Kappa coefficient values with substantial agreement (Kappa ≥ 0.61), at a minimum, red for Kappa values ≤ 0.60, and yellow for incomplete records for a given volunteer/attempt.

The data recording software was developed using JavaScript programming language, with the implementation of all the functionalities required by SB Brasil 2023. Encryption and user authentication mechanisms were incorporated to ensure the security and integrity of the data. After data collection, the information was exported to the data management system using an integrated export module that converted the data into a format compatible with statistical processing. The data management software was developed in PHP, due to its robustness and compatibility with web systems. It was designed to process the collected data, and algorithms were implemented for data validation, cleaning, and safe storage, as well as for the calculation of the agreement coefficients.

### Obtaining the agreement coefficients

The agreement coefficients were calculated both in the training and calibration phases for each condition assessed in an automated manner, using algorithms implemented in the data management system.

The overall and score-specific agreement percentages were calculated for all conditions. For posterior crossbite, which allowed binary response (presence/absence), the simple Kappa was estimated. The agreement among examiners for conditions with ordinal scale responses was evaluated using the weighted Kappa coefficient. These conditions included crown conditions, clinical consequences of untreated dental caries, canine relation, overbite, overjet in the deciduous dentition, DAI classification, and dental trauma. Specifically, regarding crown condition, the weighted Kappa coefficient was estimated, considering all codes or only those included in the calculation of the dmft/DMFT index (carious, filled with caries, filled without caries, and missing). The DAI index evaluates malocclusion according to three dimensions of dentition, spacing and occlusion based on 11 occlusal characteristics. The scores of DAI were calculated by adding the item scores, which were multiplied by their coefficients (weights). A constant is then added to the summated score. Higher values indicated worse malocclusion and greater orthodontic treatment need. DAI scores were grouped into four categories: no abnormality or minor malocclusion (DAI ≤ 25); definite malocclusion (DAI = 26–30); severe malocclusion (DAI = 31–35) and very severe malocclusion (DAI ≥ 36) according to Jenny and Cons.^16^ This classification was used for analyzing the agreement coefficients among the examiners.

The global agreement or agreement proportion observed, which is also known as the crude or raw agreement, is the simplest method for summarizing an agreement for categorical variables. It reflects the percentage of the total number of units inspected where there is agreement between the examiner and the standard examination. The agreement percentage was calculated by dividing the total number of concordant records by the total number of records.^2^ Score-specific agreement is a complement to the global agreement evaluation and is obtained by the ratio between the total number of agreements for a given score (e.g., code A for the crown condition) and the total number of examinations evaluated with that score by both examiners.

The Cohen’s kappa statistic (κ) is used to measure agreement of binary values. It is a relative measure that determines the excess of observed agreement to chance agreement. A negative value is assumed if there is complete disagreement; kappa is zero if there is no more agreement that can be expected due to chance, and one if there is perfect agreement.^2,3,17^ Weighted kappa is recommended for determining examiner reliability for ordinal data.^13^ This statistic incorporates the factor of agreement by chance alone and also weights proportional agreement. The weighted Kappa was calculated by applying linear weights, according to Cicchetti and Allison.^18^ The formulas used to calculate the weighted Kappa are described in Fleiss, Levin, and Paik.^19^

The agreement coefficients were obtained by creating cross tables between the examiner’s records and the standard examination, with row i of the table corresponding to the examiner’s records and column j to the standard examination. The formulas implemented in the data management system to obtain the weighted Kappa are presented in Table 2.

**Table 2:** 2x2 table and formulas for calculating agreement coefficients between examiners and the standard examination

*i*k*j*k	*j*_1_	*j*_2_		*j*_3_	*j*_4_	
*i*_1_	**Concordant record (i**_**1**_ **, j**_**1**_ **)**	Discordant record (*i*_1_, *j*_2_)		Discordant record (*i*_1_, *j*_3_)	Discordant record (*i*_1_, *j*_4_)	∑*j*_1_, *j*_k_
*i*_2_	Discordant record (*i*_2_, *j*_1_)	**Concordant record (i**_**2**_ **, j**_**2**_ **)**		Discordant record (*i*_2_, *j*_3_)	Discordant record (*i*_2_, *j*_4_)	∑*j*_2_, *j*_k_
*i*_3_	Discordant record (*i*_3_, *j*_1_)	Discordant record (*i*_3_, *j*_2_)		**Concordant record (i**_**3**_ **, j**_**3**_ **)**	Discordant record (*i*_3_, *j*_4_)	∑*j*_3_, *j*_k_
*i*_4_	Discordant record (*i*_4_, *j*_1_)	Discordant record (*i*_4_, *j*_2_)		Discordant record (*i*_4_, *j*_3_)	**Concordant record (i**_**4**_ **, j**_**4**_ **)**	∑*j*_4_, *j*_k_
	∑*i*_k_, *j*_1_	∑*i*_k_, *j*_2_		∑*i*_k_, *j*_3_	∑*i*k, *j*_4_	∑*i*_k_, *j*_k_
Global agreement			$\frac{Total\;sum\;of\;concordant\;records}{Total\;number\;of\;records}$			
Score-specific agreement			$\frac{Total\;records\;of\;score\;k}{Total\;number\;of\;score\;k\;records\;by\;two\;examiners}$			
Weighted Kappa						
Linear weights W_ij_ Fleiss, Levin, and Paik (2003)^19^			^ $W_{ij}=1-\frac{\left|i-j\right|}{K-1}$ ^			
Observed weighted proportion of agreement (P_ow_)			$p_{ow}=\frac{1}{n_{++}}\sum_{i}\sum_{j}w_{ij}n_{ij}$			
Chance expected weighted proportion of agreement (P_ew_)			$p_{\mathrm{ew}}={\left(\frac{1}{n_{++}}\right)}^{2}\sum_{i}\sum_{j}\left(w_{ij}n_{i+}n_{+j}\right)$			
Weighted Kappa (K_w_)			$K_{w}=\frac{\text{ Pow }-\text{ Pew }}{1-\text{ Pew }}$			
Standard error, according to Fleiss, Levin, and Paik (2003)^19^			${\hat{s}}_{0}=1/\left(\left(1-p_{e}\right)\sqrt{}n\right){\left(\left[\sum_{i}\sum_{j}p_{i}\cdot p_{.j}{\left\{w_{ij}-\left({\bar{w}}_{i.}+{\bar{w}}_{j}\right)\right\}}^{2}\right]-p_{e}^{2}\right)}^{1/2}$			
Confidence interval			$K_{w}+-z\alpha /2{\hat{e}}_{0}(k)$			

*i corresponds to the row and *j* to the column. *K* is the total number of categories of the evaluated condition, *w* corresponds to the linear weight, *n* is the total number of records.

The interpretation of the Kappa coefficient was performed according to Landis and Koch, with an examiner being considered fit for fieldwork if they achieved substantial agreement, i.e., they presented a Kappa coefficient equal to or greater than 0.61 for all the assessed conditions.^20^

### Software functionality tests

The first testing phase consisted of evaluating the software’s compatibility with the Mobile Data Collection Device (DMC) provided by the Brazilian Institute of Geography and Statistics for fieldwork, as well as checking the data entry fields, screen layout, registration process, restricted access, and data export. For this, data entry simulations were performed by five researchers, and reports were created on the necessary adjustments.

Subsequently, typing tests were conducted and data spreadsheet were generated, as well as data export to the management system, and validation of the method for calculating the agreement coefficients and demonstration in the reports. For this, data from five volunteers were entered, and the calculations were performed based on the standard examination for all the considered conditions. To validate the calculations, all kappa estimates, standard errors, and confidence intervals were performed simultaneously by a researcher in Microsoft Excel® spreadsheets and Stata® version 17 (StataCorp LLC) using the command “kap condition examiner condition_standard, wgt(w) tab”. The checks were carried out until identical values for the kappa coefficient and confidence intervals were obtained.

### In-lux calibration result

The calibration results were presented descriptively, considering the total number of examiners with records for all the assessed conditions. The minimum and maximum values for the kappa coefficient found were obtained, as well as the number of attempts required to achieve a kappa coefficient ≥ 0.61. Additionally, the distribution of examiners with substantial or excellent agreement was presented, along with the number of examiners who achieved substantial agreement in a single attempt, 2 to 4 attempts, and 5 or more attempts.

## Results

### Data recording software, demonstration of calculations, and reports

**Figure 1:**
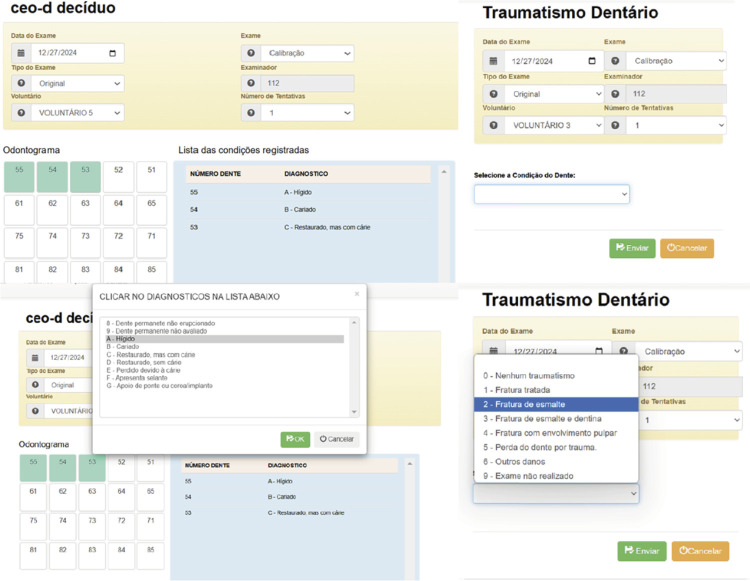
Illustrative images of the fields for recording the codes for the condition of the crown of each deciduous tooth (dmft) and dental trauma.

The software enabled the recording of codes for each condition and volunteer evaluated in pre-coded fields or in whole number entries, in the case of the DAI, through restricted access for the examiner. A dental chart was implemented for recording the crown conditions, including caries, as well as specific fields to record other conditions in the volunteers. Figure 1 illustrates the fields for recording the crown conditions of each deciduous tooth, with pre-registered options. Each set of photographs per volunteer generated 20 records, as shown in the example. The figure also illustrates the field for recording dental trauma observed in the evaluated tooth. In this case, each photograph per volunteer generated a single record (Figure 1). Examiners could complete their records in up to 10 attempts, both in the practical training and calibration phases. If the examiner, using their login and password, entered data more than once, each new entry was automatically recorded as a new attempt.

The software was created in Portuguese; therefore, the figure displays an illustrative image in this language.

**Figure 2:**
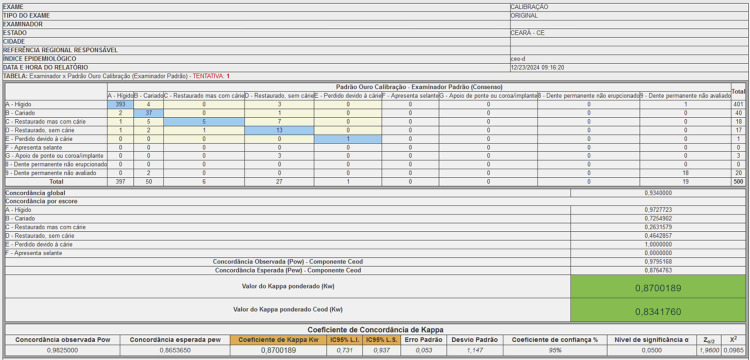
Individual report demonstrating the agreement between the examiner’s records and the standard examination for the deciduous tooth crown conditions, with the results of the overall agreement coefficients, score-specific agreement, weighted Kappa considering all categories, and weighted Kappa considering only the components of the dmft index.

The data management system generated reports for monitoring calibration by the Local References. The individual report from each examiner was issued separately for each index, presenting the cross-table between the records made by the examiner and the results of the standard examination. In the example shown in Figure 2, the individual examiner report displays the calibration results for evaluating the crown condition of a deciduous tooth (a total of 500 records). The blue diagonal shows the number of records in which there was agreement between the examiner's result and the standard examination records for the codes that constitute the dmft index. The results outside the diagonal indicate disagreement. The horizontal rows represent the examiner’s records, while the vertical rows show the results of the standard examination. For this examiner, 401 teeth were considered healthy, with 393 records agreeing with the standard examination. There were eight discordant records, of which four teeth, considered healthy by the examiner, were deemed decayed according to the standard examination. The overall agreement was 93.40%. The lowest agreement was observed for the condition “filled, but with caries” (26.31%). For this condition, of the 18 records made by the examiner, five agreed with the standard examiner. The report presents the values for the observed weighted proportion of agreement (Po_w_) and the chance-expected weighted proportion of agreement (Pe_w_). The Kw was 0.8700, considering all conditions in the crown evaluation. This value was calculated based on Po_w_ (0.9825) and Pe_w_ (0.8654), with the calculations shown in Table 3. The same calculations were repeated considering the components of the dmft index, resulting in a Kw of 0.8341.

The software was created in Portuguese; therefore, the figure displays an illustrative image in this language. The total number of records was obtained from the examination of 20 teeth in photographs of 25 volunteers.

**Table 3:** Demonstration of obtaining the K*w* coefficient for agreement between the examiner and the standard examination for the condition of the deciduous tooth crown.

Crown condition	A - Healthy	B - Decayed	C - Filled, with caries	D - Filled, without caries		E - Missing due to caries	F - Sealant	G - Fixed dental prosthesis abutment, special crown or veneer.	Unerupted permanent tooth		Unexamined permanent tooth	Total
A - Healthy	393	4	0	3		0	0	0	0		1	401
B - Decayed	2	37	0	1		0	0	0	0		0	40
C - Filled, but with caries	1	5	5	7		0	0	0	0		0	18
D - Filled, without caries	1	2	1	13		0	0	0	0		0	17
E – Missing, due to caries	0	0	0	0		1	0	0	0		0	1
F - Sealant	0	0	0	0		0	0	0	0		0	0
G - Fixed dental prosthesis abutment, special crown or veneer	0	0	0	3		0	0	0	0		0	3
Unerupted permanent tooth	0	0	0	0		0	0	0	0		0	0
Unexamined permanent tooth	0	2	0	0		0	0	0	0		18	**20**
**Total**	**397**	**50**	**6**	**27**		**1**	**0**	**0**	**0**		**19**	**500**
**Linear Weights (W**_**ij**_ **)**												
A - Healthy	1	0.875	0.75	0.625		0.5	0.375	0.25	0.125		0	
B - Decayed	0.875	1	0.875	0.75		0.625	0.5	0.375	0.25		0.125	
C - Filled, but with caries	0.75	0.875	1	0.875		0.75	0.625	0.5	0.375		0.25	
D - Filled, without caries	0.625	0.75	0.875	1		0.875	0.75	0.625	0.5		0.375	
E - Missing, due to caries	0.5	0.625	0.75	0.875		1	0.875	0.75	0.625		0.5	
F - Sealant	0.375	0.5	0.625	0.75		0.875	1	0.875	0.75		0.625	
G - Fixed dental prosthesis abutment, special crown or veneer	0.25	0.375	0.5	0.625		0.75	0.875	1	0.875		0.75	
Unerupted permanent tooth	0.125	0.25	0.375	0.5		0.625	0.75	0.875	1		0.875	
Unexamined permanent tooth	0	0.125	0.25	0.375		0.5	0.625	0.75	0.875		1	
**P**_**ow**_												
A - Healthy	393	3.5	0	1.875		0	0	0	0		0	398.375
B - Decayed	1.75	37	0	0.75		0	0	0	0		0	39.5
C - Filled, with caries	0.75	4.375	5	6.125		0	0	0	0		0	16.25
D - Filled, without caries	0.625	1.5	0.875	13		0	0	0	0		0	16
E - Missing due to caries	0	0	0	0		1	0	0	0		0	1
F - Sealant	0	0	0	0		0	0	0	0		0	0
G - Fixed dental prosthesis abutment, special crown or veneer	0	0	0	1.875		0	0	0	0		0	1.875
Unerupted permanent tooth	0	0	0	0		0	0	0	0		0	0
Unexamined permanent tooth	0	0.25	0	0		0	0	0	0		18	18.25
												**491.25**
**P**_**ew**_												
A - Healthy	159197	17543.75	1804.5	6.766.875		200.5	0	0	0		0	185.512.625
B - Decayed	13895	2000	210	810		25	0	0	0		95	17035
C - Filled, but with caries	5359.5	787.5	108	425.25		13.5	0	0	0		85.5	6779.25
D - Filled, without caries	4.218.125	637.5	89.25	459		14.875	0	0	0		121.125	5.539.875
E – Missing, due to caries	198.5	31.25	4.5	23.625		1	0	0	0		9.5	268.375
F - Sealant	0	0	0	0		0	0	0	0		0	0
G - Fixed dental prosthesis abutment, special crown or veneer	297.75	56.25	9	50.625		2.25	0	0	0		42.75	458.625
Unerupted permanent tooth	0	0	0	0		0	0	0	0		0	0
Unexamined permanent tooth	0	125	30	202.5		10	0	0	0		380	747.5
												**216341.25**
Overall agreement: $\frac{461}{500}=93.40\%$	$P_{ow}=\frac{1}{500}*491.25=0.9825$				$P_{own}=\frac{1}{500*500}*216341.25=0.865365$					$K_{w}=\frac{0.9825-0.865365}{1-0.865365}=0.8700$		

**Figure 3:**
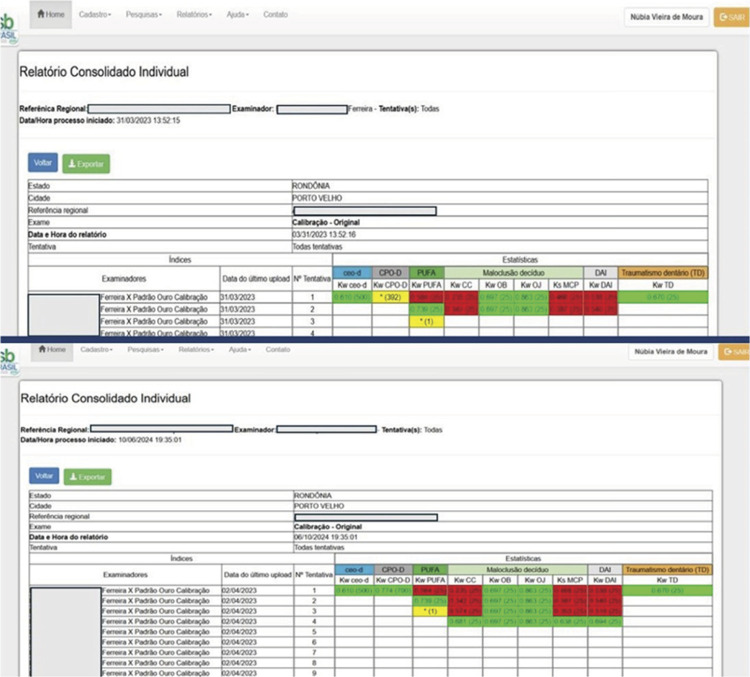
Model of an individual consolidated report with results from calibration attempts by the same examiner for all assessed conditions.

In addition to the reports separated by evaluated condition, the system generated consolidated individual reports, showing the coefficients of agreement generated in all calibration attempts made by the same examiner for all evaluated conditions/indices in a single document. In Figure 3, for example, the Kw for clinical consequences of dental caries was 0.739 in the 2^nd^ attempt. In the 1^st^ attempt, the value was 0.584 (Figure 3). For the DAI, Kappa ≥ 0.61 was obtained in the 4^th^ attempt, with the value highlighted in green.

The software was created in Portuguese; therefore, the figure displays an illustrative image in this language.

### Calibration Results

**Figure 4:**
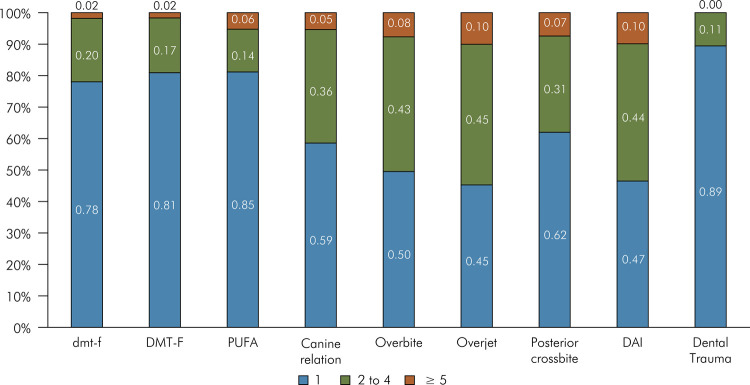
Distribution of examiners according to the number of calibration attempts until achieving substantial agreement with the standard examination (kappa ≥ 0.61).

A total of 1,513 examiners registered in the system, of which 728 (48.12%) completed calibration for all conditions and indices, with K or Kw values ≥ 0.61. The remaining registered examiners either did not have records for all indices or did not achieve substantial or higher agreement. The minimum Kappa values were 0.61 for almost all conditions, except for Posterior Crossbite, where the lowest Kappa value was 0.638. Kappa = 1 was also the maximum observed value for almost all conditions, except for the condition of the crown of deciduous teeth (dmft index). Higher percentages of examiners with almost-perfect agreement were observed for the crown condition in permanent dentition, untreated dental caries clinical consequence, and dental trauma. The highest percentage of examiners with Kappa values between 0.61 and 0.80 was observed for the DAI (Table 4). Regarding the number of attempts, occlusion conditions of deciduous and permanent dentition were those that most frequently required 2 to 4 attempts to achieve Kappa ≥ 0.61 (Figure 4).

**Table 4:** Minimum and maximum values of the Kappa coefficients obtained by the examiners for each assessed condition and the distribution of examiners according to substantial or almost-perfect agreement levels (n = 728 examiners).

Variable	Kappa coefficient		% examiners	
	Minimum value	Maximum value	Substantial agreement (Kappa 0.61 to 0.80)	Almost-perfect agreement (Kappa 0.81 to 1.00)
Crown condition in deciduous teeth (dmft) - K_w_	0.610	0.996	86.87%	13.13%
Crown condition in permanent teeth (DMFT) - K_w_	0.610	1.00	38.16%	61.84%
Clinical consequence of untreated caries - K_w_ (pufa/PUFA)	0.610	1.00	54.88%	45.12%
Canine relation - K_w_	0.610	1.00	66.55%	33.45%
Overbite - K_w_	0.610	0.936	90.51%	9.49%
Overjet - K_w_	0.610	1.00	76.35%	23.54%
Posterior crossbite – Simple Kappa	0.638	1.00	72.35%	27.65%
DAI - K_w_	0.610	1.00	91.29%	8.71%
Dental trauma - K_w_	0.610	1.00	57.72%	42.28%

## Discussion

This methodological study described the development and implementation of technological tools for the training and calibration of examiners for SB Brasil 2023. The use of the *in-lux* calibration method and a virtual learning environment (Moodle®) enabled remote training, aiming for uniformity in the application of criteria and codes for assessing oral conditions among examiners from different regions of the country. This approach represented an advance and may be particularly useful in large-scale surveys involving multiple examiners in different locations, as in this national survey.

The adoption of the *in-lux* method proved to be suitable for calibrating a large number of examiners, meeting the need to carry out the process remotely. The observed levels of agreement contributed to demonstrating the feasibility of the process, which was based on the use of a standard examination obtained through consensus and demonstrated consistency among examiners. Consensus was the strategy employed to define the standard examination, as it minimizes errors among examiners.^21^ The selection of a standard examiner is one of the main objectives of calibration, aiming to measure how far each examiner is from the standard, which is assumed to be the true value.^3^ When a standard examiner is not established, it is possible for all values observed by different examiners to be close to each other (high Kappa values) but distant from the presumed true value (that of the standard examiner). Still, in this *in-lux* calibration process, the number of photographs was defined based on WHO recommendations, which suggest that the examiner should assess at least 25 subjects to measure variations among examiners (inter-examiner reproducibility) after practicing the examination on a group of 10 subjects.^2^

The selection of volunteers for obtaining photographs was planned to anticipate the conditions that would be encountered in the field, according to the criteria of Pine, Pitts and Nugent,^22^ which is crucial in assessing consistency among examiners.^1^ This approach aims to represent the conditions to be evaluated during field research. Another important aspect is the prevalence of the condition being evaluated, which can influence the Kappa coefficient,^15,23^ the method chosen for this study as it is one of the most commonly used and robust methods to assess the reliability of measurements^24^. In this study, the average DMFT and dmft values were high, and more than 50% of the volunteers had all conditions of the index. For dental trauma and clinical consequence of dental caries, all conditions were represented in the photographs. However, for occlusal conditions, there was an absence or low prevalence of certain conditions in the primary dentition (open bite, edge-to-edge bite, anterior crossbite, and posterior crossbite). For these conditions, the most frequent was normal occlusion. Therefore, the higher number of sound volunteers (fewer diagnostic errors) compared to volunteers with occlusal alterations (more diagnostic errors) may dilute the errors attributed to occlusal alterations in the primary dentition, leading to a positive perception of the results achieved in the examiners’ calibration. However, occlusal conditions in the primary dentition and DAI required the highest number of attempts to achieve substantial agreement. This result may also reveal the difficulties in assessing these conditions using photographs. Thus, the decision to use the same set of photographs for the evaluation of crowns in both primary and permanent teeth, as well as for occlusal conditions in the primary dentition, complicated the process due to the low occurrence of certain conditions.^25^ Future studies should delve deeper into understanding the challenges associated with *in-lux* calibration using photographs specifically selected to better represent occlusal conditions in the primary dentition. Strategies that consider the use of three-dimensional images or more advanced technologies could be explored to overcome the observed limitations and enhance consistency in the evaluation of these conditions.^22^

According to WHO, where there are major discrepancies, the examiners should be re-examined so that inter-examiner differences can be reviewed and resolved by group discussion.^3^ If the variability is large, the examiner should review the interpretation of the criteria and conduct additional examinations until acceptable consistency is achieved. Otherwise, the examiner should not proceed with data collection. This aspect of calibration was implemented through the demonstration of individual reports, which were used by research teams to discuss calibration results with the examiners, reinforcing diagnostic criteria in situations with more disagreements compared to the standard examination. Thus, for some examiners it was necessary to repeat the calibration exercise two or more times. The calibration software allowed for multiple attempts to be recorded, allowing for a situation similar to “repetition” of examinations.

The functionalities developed in the software met the technical and logistical requirements of the survey, and the automation of calculations facilitated the monitoring of the calibration process by the research team. Some challenges related to the configuration of mobile data collection devices (DMCs) and internet access for data export were encountered. The desktop version aimed to overcome the app’s limitation of compatibility with only Android® operating systems. The data recorded throughout the process also contributed to generating detailed information about the calibration process, considering the number of registered examiners, the number of examiners with incomplete records, examiners who did not achieve the minimum agreement required for fieldwork, agreement coefficient values, among others. This study focused on presenting the Kappa index, as it was employed to determine whether or not the examiner could continue fieldwork. Future publications should analyze overall agreement indices and score-specific agreement, along with their relationship with the Kappa index, aiming to further detail the main discrepancies.

By integrating photographic records and specialized software, this innovative calibration process offers a scalable solution for diverse and challenging contexts, ensuring methodological consistency and technological efficiency. It is believed that this study contributes to strengthening oral health surveillance in Brazil by promoting standardized methodologies for data collection. It is expected that the experiences shared in this study may support future epidemiological surveys, with enhancements to address the challenges encountered.

A limitation of the calibration process in SB Brasil 2023 was the absence of duplicate evaluations to assess intra-examiner consistency over time. Without these evaluations, it was not possible to verify whether examiners maintained consistent application of diagnostic criteria throughout the data collection period. This step was not carried out as it proved to be unfeasible during data collection, given the difficulty of obtaining subjects’ authorization to conduct a second examination. Additionally, for occlusal conditions, the high prevalence of volunteers without alterations may have diluted diagnostic errors, creating an optimistic perception of inter-examiner agreement for these conditions. Despite the limitations, the technological tools implemented in SB Brasil 2023 enabled online training and calibration, allowing for the attainment of examiner consistency for fieldwork. These results highlight the feasibility of remote strategies for calibration in epidemiological surveys, particularly in scenarios involving multiple geographically distributed examiners, with potential applications in other contexts and health fields.

## References

[1] Assaf AV, Tagliaferro EP, Meneghim MC, Tengan C, Pereira AC, Ambrosano GM (2007). A new approach for interexaminer reliability data analysis on dental caries calibration. J Appl Oral Sci.

[2] World Health Organization (2013). Oral health surveys: basic methods.

[3] World Health Organization (1993). Calibration of examiners for oral health epidemiology surveys.

[4] Christian B, Amezdroz E, Calache H, Gussy M, Sore R, Waters E (2017). Examiner calibration in caries detection for populations and settings where in vivo calibration is not practical. Community Dent Health.

[5] Andrade FR, Narvai PC, Montagner MA (2016). The ethics of in vivo calibrations in oral health surveys. Rev Bras Epidemiol.

[6] Martins AM, Silveira MF, Freitas CV, Eleutério NB, Oliveira PH, Ferreira RC (2011). Desafios de um exercício de calibração para estudo epidemiológico envolvendo variáveis quantitativas e categóricas ordinais. Arq Odontol.

[7] Pinto RD, Vettore MV, Abreu MH, Palmier AC, Moura RN, Roncalli AG (2023). Reliability analysis using the in-lux examination method for dental indices in adolescents for use in epidemiological studies. Community Dent Oral Epidemiol.

[8] Ministério da Saúde (BR), Secretaria de Vigilância em Saúde, Secretaria de Atenção à Saúde, Departamento de Atenção Básica, Coordenação Nacional de Saúde Bucal (2011). SB Brasil 2010: Pesquisa Nacional de Saúde Bucal: resultados principais.

[9] Pinto RS, Lopes DL, Santos JS, Roncalli AG, Projeto SB (2018). Minas Gerais 2012: pesquisa das condições de saúde bucal da população mineira: métodos e resultados principais. Arq Odonto.

[10] Goettems ML, Correa MB, Vargas-Ferreira F, Torriani DD, Marques M, Domingues MR (2013). Methods and logistics of a multidisciplinary survey of schoolchildren from Pelotas, in the Southern Region of Brazil. Cad Saude Publica.

[11] Felsch M, Meyer O, Schlickenrieder A, Engels P, Schönewolf J, Zöllner F (2023). Detection and localization of caries and hypomineralization on dental photographs with a vision transformer model. NPJ Digit Med.

[12] Avenetti DM, Martin MA, Gansky SA, Ramos-Gomez FJ, Hyde S, Van Horn R (2023). Calibration and reliability testing of a novel asynchronous photographic plaque scoring system in young children. J Public Health Dent.

[13] Cohen J (1968). Weighted kappa: nominal scale agreement with provision for scaled disagreement or partial credit. Psychol Bull.

[14] Ministério da Saúde (BR) (2022). SB Brasil 2020: Pesquisa Nacional de Saúde Bucal: projeto técnico.

[15] Agbaje JO, Mutsvari T, Lesaffre E, Declerck D (2012). Measurement, analysis and interpretation of examiner reliability in caries experience surveys: some methodological thoughts. Clin Oral Investig.

[16] Jenny J, Cons NC (1996). Establishing malocclusion severity levels on the Dental Aesthetic Index (DAI) scale. Aust Dent J.

[17] Cohen J (1960). A coefficient of agreement for nominal scales. Educ Psychol Meas.

[18] Cicchetti DV, Allison T (1971). A new procedure for assessing reliability of scoring EEG sleep recordings. Am J EEG Technol.

[19] Fleiss JL, Levin B, Paik MC (2003). Statistical methods for rates and proportions.

[20] Landis JR, Koch GG (1977). The measurement of observer agreement for categorical data. Biometrics.

[21] Frias AC, Antunes JL, Narvai PC (2004). Precisão e validade de levantamentos epidemiológicos em saúde bucal: cárie dentária na Cidade de São Paulo, 2002. Rev Bras Epidemiol.

[22] Pine CM, Pitts NB, Nugent ZJ (1997). British Association for the Study of Community Dentistry (BASCD) guidance on the statistical aspects of training and calibration of examiners for surveys of child dental health: a BASCD coordinated dental epidemiology programme quality standard. Community Dent Health.

[23] Shankar V, Bangdiwala SI (2014). Observer agreement paradoxes in 2x2 tables: comparison of agreement measures. BMC Med Res Methodol.

[24] McHugh ML (2012). Interrater reliability: the kappa statistic. Biochem Med (Zagreb).

[25] Agbaje JO, Mutsvari T, Lesaffre E, Declerck D (2012). Examiner performance in calibration exercises compared with field conditions when scoring caries experience. Clin Oral Investig.

